# Dynamics of multipartite quantum steering for different types of decoherence channels

**DOI:** 10.1038/s41598-023-30869-5

**Published:** 2023-03-07

**Authors:** Wei-Chen Li, Ya Xiao, Xin-Hong Han, Xuan Fan, Xiao-Bing Hei, Yong-Jian Gu

**Affiliations:** College of Physics and Optoelectronic Engineering, Ocean University of China, Qingdao, 266100 People’s Republic of China

**Keywords:** Quantum information, Qubits

## Abstract

Multipartite quantum steering, a unique resource for asymmetric quantum network information tasks, is very fragile to the inevitable decoherence, which makes it useless for practical purposes. It is thus of importance to understand how it decays in the presence of noise channels. We study the dynamic behaviors of genuine tripartite steering, reduced bipartite steering, and collective steering of a generalized three-qubit W state when only one qubit interacts independently with the amplitude damping channel (ADC), phase damping channel (PDC) or depolarizing channel (DC). Our results provide the region of decoherence strength and state parameters that each type of steering can survive. The results show that these steering correlations decay the slowest in PDC and some non-maximally entangled states more robust than the maximally entangled ones. Unlike entanglement and Bell nonlocality, the thresholds of decoherence strength that reduced bipartite steering and collective steering can survive depend on the steering direction. In addition, we find that not only one party can be steered by a group system, but also two parties can be steered by a single system. There is a trade-off between the monogamy relation involving one steered party and two steered parties. Our work provides comprehensive information about the effect of decoherence on multipartite quantum steering, which will help to realize quantum information processing tasks in the presence of noise environments.

## Introduction

Quantum steering^1^ enables an untrusted party to remotely steer the quantum state of other trusted parties by performing local measurement on his own state. It was first put forward by Schrödinger^2,3^ as a reply to the well-known EPR paradox^4^. However, it did not attach much attention until 2007 when Wiseman et al. provided an operational definition and specific experimental criteria for quantum steering based on the local hidden state model in the form of quantum information tasks^5,6^. According to the hierarchy of quantum correlations, quantum steering stands between entanglement and Bell nonlocality. Especially, it exhibits unique asymmetric feature, which can lead to one-way steering where one party can steer the other party’s state, but not vice versa^7–9^. The asymmetric steering has been experimentally demonstrated both in continuous and discrete variable bipartite systems^10–13^.

Another directional feature of quantum steering is the monogamy relation in multipartite system. Reid found that one party can steer two independent systems, but it is impossible for two parties to independently demonstrate steering of a third system^14^. There are more various steering scenarios in multipartite system compared with the bipartite steering. For example, in the tripartite system, there are genuine tripartite steering, reduced bipartite steering and collective steering^1^. In addition, multipartite steering can be further applied to large-scale quantum networks^15^. These properties make multipartite steering has received increasing attention. There has been abundant research on multipartite steering: various criteria for multipartite steering have been developed and verified in experiments with different systems^16,17^; how shared steering can be distributed among different parties has been investigated^18–22^; methods for super-activating steering have been proposed^23^; sequential detection of genuine tripartite steering via unsharp measurements has been demonstrated^24^, etc.

As for applications, quantum steering has been identified as a unique physical resource^25^ for one-sided device-independent quantum key distribution^26–28^, subchannel discrimination^29–31^, quantum secret sharing^32,33^, quantum teleportation^34–36^, and randomness certification^37,38^. Therefore, it is essential to preserve the quantum steering in a quantum system. However, the quantum system inevitably interacts with its surroundings which can be described as different decoherence channels. As a result, the system may lose its quantum properties partially or completely (known as sudden death), rendering it unusable for quantum information tasks. Up to now, the effects of decoherence channels on entanglement^39–41^, Bell nonlocality^42,43^, coherence^44,45^ and discord^46,47^ have been widely studied both theoretically and experimentally. From a practical point of view, it is necessary to study the dynamical behavior of quantum steering in the real world.

Recently, the loss in quantum channels has been studied in verifying quantum steering^48–50^, with the sudden death of quantum steering taken into consideration^51^. The effects of decoherence on entanglement, steering and Bell nonlocality have been compared^52,53^. Besides, weak measurement, distillation, non-Markovian environment, and correlated channel are proved to be effective for recovering the disappeared steerability^53–57^.

However, all of these works are limited to bipartite system, the decoherence effect on multipartite quantum steering is still missing. In this work, we take the generalized three-qubit W state as an example, investigating the decay dynamics of multipartite steering when only one party transmits through amplitude damping channel (ADC), phase damping channel (PDC) and depolarizing channel (DC), respectively. First, we provide the region of decoherence strength and state parameters that the genuine tripartite steering can survive in one-sided device-independent (1SDI) and two-sided device-independent (2SDI) scenarios, then compare them with genuine tripartite entanglement and genuine tripartite Bell nonlocality. The results show that as the decoherence increases, entanglement, 1SDI steering, 2SDI steering and Bell nonlocality decay faster in turn, and they exhibit distinct decaying behaviors in different channels. Interestingly, we find that when one of the state parameters is small, the area of the steerable region of the other state parameters and decoherence strength in the ADC is almost the same as that in the PDC, while they are almost the same in the ADC and DC when the state parameter is large. Furthermore, by reconstructing steering parameters involving average inference variance for various bipartite splits, we find that it is possible to manipulate the direction of reduced bipartite steering, even the symmetry of collective steering, via changing the decoherence strength. The results also show collective steering is more robust than reduced bipartite steering. We further present how to manipulate the distribution of steerability among different parties through decoherence. These results provide a useful reference for applying quantum steering in decoherence environments and promoting the development of quantum steering-based quantum information technologies.

## Results

### Channels models and state dynamics

We start by describing the channel models and their effects on the evolution of system state. Quantum channel is a completely positive trace-preserving map which has a representation in terms of Kraus operators $\lbrace K_{m} \rbrace$, where $\sum _{m} K_{m}^{\dagger }K_{m}=I$ and *I* is a identity matrix. In the present study, we restrict ourselves to three typical noise channels, viz. ADC, PDC and DC. The corresponding Kraus operators of these channels, with the strength of decoherence *d*, are shown in Table 1. The inevitable interaction between system and noise channels will give rise to a decay of system correlation. To fully investigate the dissipative dynamic of steerability in multipartite system, we take the generalized three-qubit W state $\vert \psi _{gW}\rangle =\alpha \vert 001\rangle +\beta \vert 010\rangle +\sqrt{1-\alpha ^2-\beta ^2}\vert 100\rangle$ as an illustration, where $\alpha$, $\beta \in [0,1]$^58–60^.

**Table 1:** Kraus operators for the corresponding ADC, PDC and DC, where $d\in [0,1]$ represents the decoherence strength.

Channels	Kraus operators
ADC	$K_{0}=\vert 0\rangle \langle 0\vert +\sqrt{1-d}\vert 1\rangle \langle 1\vert$, $K_{1}=\sqrt{d}\vert 0\rangle \langle 1\vert$.
PDC	$K_{0}=\vert 0\rangle \langle 0\vert +\sqrt{1-d}\vert 1\rangle \langle 1\vert$, $K_{1}=\sqrt{d}\vert 1\rangle \langle 1\vert$.
DC	$$\begin{array}{ll}K_{0}=\sqrt{1-3d/4}I,&{} K_{1}= \sqrt{d/4}\sigma _{x}, \\ K_{2}= \sqrt{d/4}\sigma _{y},&{} K_{3}= \sqrt{d/4}\sigma _{z} \end{array}$$

$\{\sigma _{x},\sigma _{y},\sigma _{z}\}$ represent Pauli operators.

Consider the case where a three-qubit state $\rho _{ABC}=\vert \psi _{gW}\rangle \langle \psi _{gW}\vert$ is initially shared among three space-like separated parties, say, Alice, Bob, and Charlie. For simplicity, assuming that only Charlie’s qubit interacts with the environment simulated by a noise quantum channel of decoherence strength *d*, the state becomes^61^:

1 $$\begin{aligned} \varepsilon (\rho _{ABC})=\sum _{m=1}^{n}(I^{A}\otimes I^{B}\otimes K_{m}^{C})\rho _{ABC}(I^{A}\otimes I^{B}\otimes K_{m}^{C})^\mathrm {\dagger }, \end{aligned}$$

where $I^{A}$ and $I^{B}$ represent the identity matrix on Alice’s and Bob’s side, respectively. The value of *n* depends on the number of Kraus operators characterizing a channel. Replacing Kraus operators given by Table 1, the corresponding elements of density matrix $\varepsilon (\rho _{ABC})$ can be obtained, see Supplementary Information for more details. In the following, we intend to study the dependence of genuine tripartite steerability, reduced bipartite steerability and collective steerability of state $\varepsilon (\rho _{ABC})$ on the decoherence strength.

### Dynamical behaviors of genuine tripartite steering

#### Detection of genuine tripartite steering

There are two different approaches for defining genuine multipartite steering. The first approach sees steering as a semi-device independent entanglement verification in the multipartite scenario where the untrusted party is fixed^18^. The second approach uses steering between the bipartitions to define genuine multipartite steering where each party is sometimes trusted and sometimes untrusted^19^. Here, we adopted the definition of genuine multipartite steering where the untrusted party is fixed.

In this section, we study the effects of ADC, PDC, and DC on genuine tripartite steering. Here two different scenarios may arise: First, only one party’s measurement device is untrusted, which is known as 1SDI scenario. Secondly, only two parties’ measurement devices are untrusted, i.e, 2SDI scenario.

In the 1SDI scenario, without loss of generality, one can consider the case where Alice tries to genuinely steer Bob and Charlie, i.e., the measurement device on Alice’s side is untrusted, while Bob’s and Charlie’s are trusted.

Similarly, in the 2SDI scenario, one can consider the case that Alice and Bob try to genuinely steer Charlie, that is, the measurement devices on Alice’s and Bob’s side are untrusted, while the device on Charlie’s side is trusted.

Here, we adopt the experimentally testable inequality $$W_{1} $$ and $$ W_{2}$$ for demonstrating genuine tripartite steering from Alice to Bob and Charlie and the steering from Alice and Bob to Charlie, respectively (see more details in the “Methods” section).

#### Effects of decoherence channels on genuine tripartite steering

**Figure 1:**
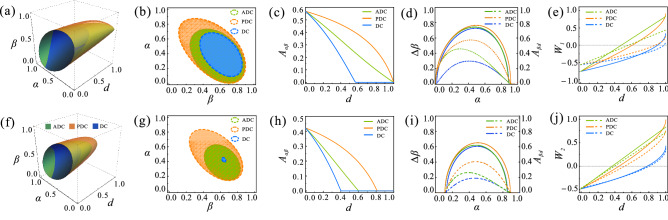
Upper panel: The effects of different decoherence channels on the dynamics of 1SDI genuine tripartite steering. (**a**) The steerable regions are parameterized by decoherence strength *d* and state parameters $\lbrace \alpha$, $\beta \rbrace$. The regions enclosed by the green, orange, and blue surfaces correspond to the existence of 1SDI genuine tripartite steering in ADC, PDC, and DC, respectively. (**b**) The sectional views of (**a**) when $d=0.4$. (**c**) The area of steerable state parameters $A_{\alpha \beta }$ as a function of decoherence strength *d*. (**d**) The maximum steerable state parameter range $\Delta \beta$ and the area of steerable region $A_{\beta d}$ as a function of state parameter $\alpha$. (**e**) The 1SDI genuine tripartite steering parameter $W_{1}$ as a function of decoherence strength *d*. Bottom panel: same as upper column but in the case of 2SDI scenario. The results of ADC, PDC, and DC are displayed in green, orange, and blue in turn. The solid lines and dashed lines shown in (**d,i**) correspond to $\Delta \beta$ and $A_{\beta d}$, respectively. The solid lines and dashed lines shown in (**e**) correspond to $\alpha =\beta =1/\sqrt{3}$ and $\alpha =3/10$, $\beta =7/10$, respectively. The solid lines and dashed lines shown in (**j**) correspond to $\alpha =\beta =1/\sqrt{3}$ and $\alpha =1/2$, $\beta =3/5$, respectively.

We investigate the dynamical behaviors of genuine tripartite steering by changing the input state parameters $\lbrace \alpha$, $\beta \rbrace$ and decoherence strength *d* of different decoherence channels. The steerabilities in the 1SDI scenario and 2SDI scenario are tested by violating the inequalities (3) and (4). The corresponding results are respectively depicted in Fig. 1a–j. Clearly, the effects of decoherence channels on genuine tripartite steering in both scenarios are similar. Figure 1a,f show the effective ranges of $\lbrace \alpha$, $\beta$, $d\rbrace$ for genuine tripartite steering. The green, orange, blue surfaces represent the steerable boundaries, in turn, indicating the existence of quantum steering under ADC, PDC and DC. Fig. 1b,g are sectional views of Fig. 1a,f when $d=0.4$. Clearly, in both scenarios, it is in the PDC that the number of quantum states whose steerability can survive is the largest. It means the genuine tripartite steering is the most robust in the PDC. To further support this conclusion, we investigate the relation between the area of the steerable state parameters $A_{\alpha \beta }$ and decoherence strength *d*. As shown in Fig. 1c,h, $A_{\alpha \beta }$ decreases the slowest in the PDC and the fastest in the DC with the increase of *d*. Clearly, in the 1SDI scenario, the genuine tripartite steerability decays to zero at $d=1$ under ADC and PDC, but in the 2SDI scenario, it disappears completely at $d<1$, regardless of the type of decoherence channels. This is because that there are more untrusted measurement devices in the 2SDI scenario, and more residual correlations are need to verify the existence of steerability.

To clarify the dependence of the robustness of genuine tripartite steering on the initial shared state $$\rho _{ABC} $$, we further calculate the maximum steerable range $$\Delta \beta $$ of state parameter $$\beta $$ and the area of the steerable region $$A_{\beta d}$$ by changing the other state parameter $$\alpha $$. The relations between $$ \Delta \beta $$ ($$A_{\beta d}$$ ) and $$ \alpha $$ in 1SDI scenario and 2SDI scenario are shown in Fig. 1d,i, respectively. In the 1SDI scenario, the birth of genuine tripartite steering occurs at approximately $$ \alpha =0 $$ and the death of that occurs around $$ \alpha =0.92 $$. While, in the 2SDI scenario, the value of $$\alpha $$ that causes the birth of genuine tripartite steering increases to $$ \alpha =0.11 $$ and the value that causes the death of genuine tripartite steering decreases to $$ \alpha =0.89 $$. This again demonstrates that the genuine tripartite steering is more robust to decoherence in the 1SDI scenario. Interestingly, we find that, both in the 1SDI scenario and 2SDI scenario, when the parameter $$\alpha $$ is small, the steerable area $$ A_{\beta d}$$ and the steerable range $$\Delta \beta $$ in the ADC are almost the same as those in the PDC, while when $$\alpha $$ is large, they are almost the same in the ADC and DC. Note that due to the asymmetric nature of these decoherence channels, the dynamic of genuine tripartite steering does not show a symmetric behavior with respect to $$ \alpha =1/\sqrt{3} $$. Counter-intuitively, the strength of decoherence that the genuine tripartite steering can survive is not the largest even when $$\rho _{ABC} $$ is maximally entangled. As shown in Fig. 1e,j, whether under the ADC, PDC or DC, the genuine tripartite steering parameters $$W_{1}$$ with $$\alpha =3/10$$, $$\beta =7/10$$ and $$ W_{2}$$ with $$\alpha =1/2$$, $$\beta =3/5$$ decays slower than that of the state with $$\alpha =\beta =1/\sqrt{3}$$.

In addition, it is interesting to compare the decay phenomena among various nonlocal quantum correlations of entanglement, steering and Bell nonlocality. The inequality suitable for detecting genuine entanglement and the Svetlichny inequality for testing genuine Bell nonlocality of the three-qubit W state are shown in the section of Methods.

**Figure 2:**
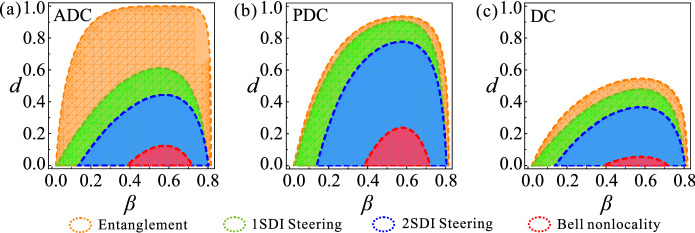
The dynamics of various genuine tripartite nonlocal quantum correlations under ADC (**a**), PDC (**b**), and DC (**c**). The regions enclosed by the orange dashed curves, green dashed curves, blue dashed curves and red dashed curves correspond to the existence of genuine tripartite entanglement, 1SDI genuine tripartite steering, 2SDI genuine tripartite steering, and genuine tripartite Bell nonlocality, respectively.

We present region plots of different genuine nonlocal quantum correlations of the state $\varepsilon (\rho _{ABC})$ with $\alpha =1/\sqrt{3}$ with respect to the decoherence strength *d* and the other state parameter $\beta$ in Fig. 2. The regions enclosed by the orange dashed curves, green dashed curves, blue dashed curves and red dashed curves correspond to the existing of genuine tripartite entanglement, 1SDI genuine tripartite steering, 2SDI genuine tripartite steering, and genuine tripartite Bell nonlocality, respectively. Clearly, genuine tripartite steering and genuine tripartite Bell nonlocality decay the slowest in the PDC, while the genuine tripartite entanglement decay the slowest in the ADC. Besides, Genuine tripartite entanglement is the most robust to decoherence, and genuine tripartite Bell nonlocality is the least robust to decoherence, as well as the robustness of 1SDI or 2SDI genuine tripartite steering is between them. These results again demonstrate the relationship between nonlocal quantum correlations as Bell nonlocality $\subset$ 2SDI steering $\subset$ 1SDI steering $\subset$ entanglement. Clearly, the maximum decoherence strength at which the corresponding genuine nonlocal quantum correlation can survive is obtained with different initial states, and they become asymmetrical with respect to $\beta$ after ADC, PDC and DC. This happens because ADC, PDC and DC do not affect $\vert 0 \rangle$ and $\vert 1 \rangle$ symmetrically.

### Dynamical behaviors of reduced bipartite steering

#### Detection of reduced bipartite steering

This section studies the effects of ADC, PDC and DC on the reduced bipartite steering. The reduced states between Alice and Bob, Alice and Charlie, Bob and Charlie can be obtained by taking the partial trace of the density matrix, as is shown in Eq. (1), i.e., $\varepsilon (\rho _{AB}) =\text{Tr}_{C}[\varepsilon (\rho _{ABC})]$, $\varepsilon ( \rho _{AC}) =\text{Tr}_{B} [\varepsilon (\rho _{ABC})]$, and $\varepsilon (\rho _{BC}) =\text{Tr}_{A}[\varepsilon (\rho _{ABC})]$, see more details in the Supplementary Information. Here, we employ a steering criterion based on average inference variance to test the steerability from party *i* to party *j* (see more details in the section of “Methods” section and in the Supplementary Information).

#### Effects of decoherence channels on reduced bipartite steering

**Figure 3:**
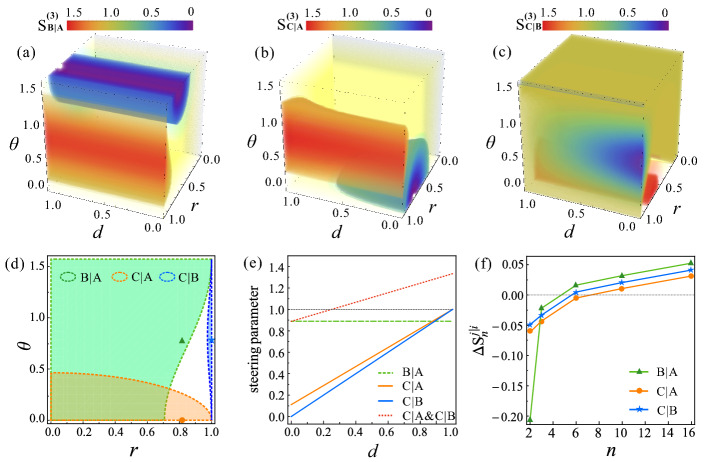
The dynamics of reduced bipartite steering under PDC. (**a–c**) The steering parameters $$ S^{(3)}_{B\vert A} $$, $$S^{(3)}_{C\vert A} $$ and $$ S^{(3)}_{C\vert B}$$ as functions of decoherence strength *d* and state parameters $$ \lbrace r, \theta \rbrace $$, respectively. (**d**) The reduced bipartite steering regions are parameterized by $$ \lbrace r, \theta \rbrace $$ for a fixed $$ d=9/10 $$. The green region enclosed by dashed green line represents Alice can steer Bob, the orange region enclosed by dashed orange line represents Alice can steer Charlie, and the blue region enclosed by dashed blue line represents Bob can steer Charlie. (**e**) The steering parameters $$ S^{(3)}_{B\vert A} (r=\sqrt{2/3}, \theta =\pi /4)$$ (green dashed line), $$ S^{(3)}_{C\vert A} (r=\sqrt{2/3}, \theta =0)$$ (orange line), $$ S^{(3)}_{C\vert B}(r=1, \theta =\pi /4)$$ (blue line) and $$ S^{(3)}_{C\vert B( A)}(r=\sqrt{2/3}, \theta =\pi /4)$$ (red dashed line) as a function of *d*. (**f**) The difference between $$S_{n}^{j\vert i}$$ and $$ C_{n} $$ ($$\Delta S_{n}^{j\vert i}$$) for the three reduced bipartite states marked as green triangle ($$ r=\sqrt{2/3}, \theta =\pi /4 $$), orange circle ($$r=\sqrt{2/3},\theta =0$$), blue star ($$r=1, \theta =\pi /4$$) in (**d**) for a fixed $$d=9/10 $$. $$S_{n}^{j\vert i}>C_{n} $$ indicates the existence of the corresponding reduced bipartite steering, where $$i\in \lbrace A, B\rbrace $$, $$j\in \lbrace B, C\rbrace $$.

Since the dynamics of reduced bipartite steering under ADC, PDC and DC are similar, and the steerability is the most robust under the PDC of 1SDI scenario, we focus on investigating the effect of PDC on the reduced bipartite steering in the 1SDI scenario. For convenience, we define $r=\sqrt{\alpha ^{2}+\beta ^{2}}$, $\theta = \text{arccot} (\alpha /\beta )$. The steering parameters $S^{(3)}_{B\vert A}$, $S^{(3)}_{C\vert A}$ and $S^{(3)}_{C\vert B}$ as functions of decoherence strength *d* and state parameters $\lbrace r, \theta \rbrace$ are shown in Fig. 3a–c, respectively. Clearly, $S^{(3)}_{B\vert A}$ is independent of *d*, and the steerability from Alice to Bob is only affected by $\lbrace r, \theta \rbrace$. This is because $\varepsilon (\rho _{AB})$ is regardless of *d*. However, as *d* increases, both $S^{(3)}_{C\vert A}$ and $S^{(3)}_{C\vert B}$ decrease. As shown in Fig. 3d, for a particular decoherence strength, such as $d=9/10$, the region of $\lbrace r, \theta \rbrace$ enabling $S^{(3)}_{C\vert A}<1$ (enclosed by orange dashed line) is larger than that enabling $S^{(3)}_{C\vert B}<1$ (enclosed by blue dashed line). This means the steerability from Alice to Charlie is more robust than from Bob to Charlie. Interestingly, for some initial states, such as $\varepsilon (\rho _{AB})$ with $r<\frac{1}{2}\sqrt{3-\cos (2\theta )}$, $\varepsilon (\rho _{AC})$ with $\theta =0$ and $\varepsilon (\rho _{BC})$ with $r=1$, the corresponding reduced bipartite steerability can survive as long as $d<1$. To clarify this, we show the results for the reduced bipartite states marked as green triangle ($r=\sqrt{2/3}, \theta =\pi /4$), orange circle ($r=\sqrt{2/3},\theta =0$), blue star ($r=1, \theta =\pi /4$) in Fig. 3d. The steering parameters $S^{(3)}_{B\vert A} (r=\sqrt{2/3}, \theta =\pi /4)$ (green dashed line), $S^{(3)}_{C\vert A} (r=\sqrt{2/3},\theta =0)$ (orange line), and $S^{(3)}_{C\vert B}(r=1, \theta =\pi /4)$ (blue line) as a function of *d* are shown in Fig. 3e. Clearly, when the initial shared tripartite state $\rho _{ABC}$ is maximally entangled, i.e. $r=\sqrt{2/3}$, $\theta =\pi /4$, the steerability from Alice to Charlie and that from Bob to Charlie completely disappear at $d=1/4$ (red dashed line), which decays faster than $S^{(3)}_{C\vert A} (r=\sqrt{2/3},\theta =0)$ and $S^{(3)}_{C\vert B}(r=1, \theta =\pi /4)$.

To illustrate the advantages of the average conditional variance-based steering criterion, we further consider the ability of party *i* to steer party *j* by using the *n*-setting linear steering inequality, which is represented as $$ S_{n}^{j\vert i}\equiv \frac{1}{n}\sum _{m=1}^{n}\langle {\sigma _{m}^{i}\sigma _{m}^{j}}\rangle \le C_{n} $$^62,63^. $$C_{n}$$ is the maximum value of $$S_{n}^{j\vert i}$$ when party *j*’s system can be described by a local hidden state model. Figure 3f presents the difference between $$S_{n}^{j\vert i}$$ and $$ C_{n} $$ ($$\Delta S_{n}^{j\vert i}=S_{n}^{j\vert i}-C_{n}$$) for the reduced bipartite states marked as green triangle, orange circle, blue star in Fig. 3d for a fixed $$d=9/10 $$. If $$\Delta S_{n}^{j\vert i}>0 $$, the steerability is demonstrated. We find that the number of the measurement settings for $$\Delta S_{n}^{j\vert i}>0 $$ is more than three, and when $$ n=3 $$, the corresponding reduced bipartite steerabilities shown in Fig. 3e have not disappeared completely, which means the steering criterion based on average inference variance is more efficient.

**Figure 4:**
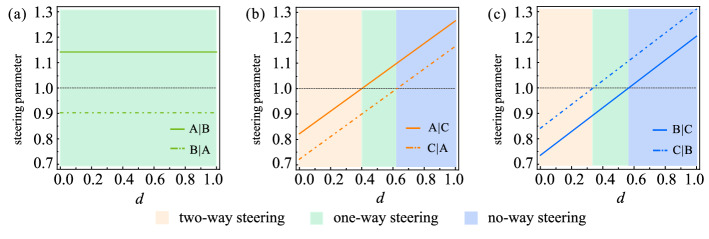
The effects of decoherence channels on the direction of reduced bipartite steering. (**a**) The steerability of $\varepsilon (\rho _{AB})$ with $r=1/\sqrt{3}$, $\theta =\pi /6$ vs *d*. (**b**) The steerability of $\varepsilon (\rho _{AC})$ with $r=1/\sqrt{3}$, $\theta =\pi /4$ vs *d*. (**c**) The steerability of $\varepsilon (\rho _{BC})$ with $r=8/9$, $\theta =\pi /6$ vs *d*. Orange, green, and blue regions correspond to two-way steerable, one-way steerable and no-way steerable, respectively.

We also study the effect of PDC on the steering direction. The results are shown in Fig. 4. Obviously, with an appropriate *d*, the one-way steering between Alice and Bob, Alice and Charlie, as well as Bob and Charlie can be observed simultaneously. And it is clear that a tunable *d* allows the states $\varepsilon (\rho _{AC})$ and $\varepsilon (\rho _{BC})$ to be shifted from a region where it is two-way steerable to an one-way steerable region, finally, to a region where it is unsteerable in both directions. Clearly, as *d* increases, the steerability from party *i* to party *j* decreases at the same rate as that from party *j* to party *i*, see Supplementary Information for details.

### Dynamical behaviors of collective steering

#### Detection of collective steering

In addition to the reduced bipartite steering mentioned above, there is another bipartite steering scenario in the tripartite system, known as collective steering, where one party can be steered collectively by a group of other two parties, but not by any individual of them^14,19^. It is well known that quantum steering exhibits a monogamous relationship^19–22^. Collective steering, however, shows that quantum steering can also exhibit polygamous properties. This feature thus opens the possibility for the realization of quantum secret sharing^32,33^ and quantum key authentication^64^. Numerous collective steering criteria have been derived, but mostly refer to the continuous-variable system^65,66^.

Here, we extend the average inference variance-based two-qubit steering criterion into collective steering by replacing single steering party with steering groups. Then, we can obtain the *n* -setting steerability parameter from the group parties *i* and *j* to party *k*, see more details in the section of Methods.

#### Effects of decoherence channels on collective steering

**Figure 5:**
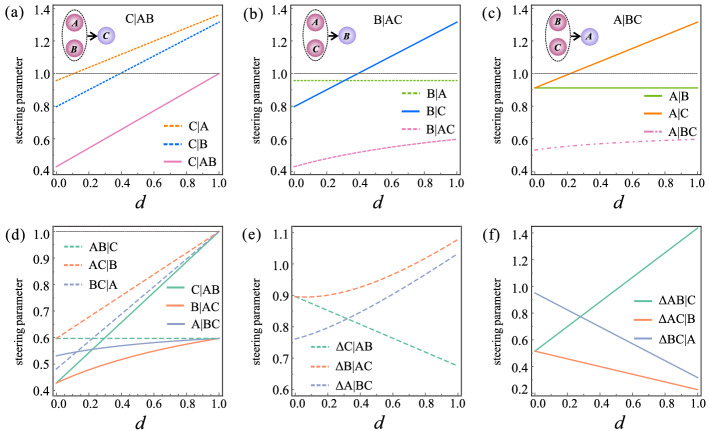
The effects of decoherence channels on the steerability between single party and group parties when $r=3\sqrt{2}/5$, $\theta =\pi /4$. (**a**) The steering parameters $\lbrace S_{C\vert \lbrace AB\rbrace }^{(3)}, S_{C\vert A}^{(3)}, S_{C\vert B }^{(3)}\rbrace$ for Charlie being steered party as a function of *d*. (**b**) The steering parameters $\lbrace S_{B\vert \lbrace AC\rbrace }^{(3)}, S_{B\vert A}^{(3)}, S_{B\vert C }^{(3)}\rbrace$ for Bob being steered party as a function of *d*. (**c**) The steering parameters $\lbrace S_{A\vert \lbrace BC\rbrace }^{(3)}, S_{A\vert B}^{(3)},$ $S_{A\vert C }^{(3)} \rbrace$ for Alice being steered party as a function of *d*. (**d**) The effects of decoherence on the direction of collective steering. (**e**) The monogamy relation for one steered party. (**f**) The monogamy relation for two steered parties. $S_{k\vert \lbrace ij\rbrace }^{(3)}<1$, $S_{\lbrace ij\rbrace \vert k}^{(3)}<1$ and $S_{k\vert i(j) }^{(3)}<1$ indicate the existence of the corresponding types of steering, $\Delta S_{k\vert \lbrace ij\rbrace }^{(3)}>0$ and $\Delta S_{\lbrace ij\rbrace \vert k}^{(3)}>0$ indicate the satisfaction of the corresponding monogamy relation, where $i\in \lbrace A, B\rbrace$, $j\in \lbrace B,C\rbrace$, $k\in \lbrace A,B,C\rbrace$.

Since the decay behaviors of collective steering under ADC, PDC and DC are similar, here we focus on the case of PDC in the 1SDI scenario. We compare the dynamics of collective steering and its corresponding reduced bipartite steering, the results of the state $\varepsilon (\rho _{ABC})$ with $r=3\sqrt{2}/5$, $\theta =\pi /4$ are shown in Fig. 5. The steering parameter groups $\lbrace S_{C\vert \lbrace AB\rbrace }^{(3)}, S_{C\vert A}^{(3)}, S_{C\vert B }^{(3)}\rbrace$, $\lbrace S_{B\vert \lbrace AC\rbrace }^{(3)}, S_{B\vert A}^{(3)}, S_{B\vert C }^{(3)}\rbrace$ and $\lbrace S_{A\vert \lbrace BC\rbrace }^{(3)}, S_{A\vert B}^{(3)}, S_{A\vert C }^{(3)} \rbrace$ as a function of *d* are presented in Fig. 5a–c, respectively. Clearly, with the increase of decoherence strength, the decay speed of the steerabilities between Alice and Bob (green lines), between Bob and Charlie (blue line), between Alice and Charlie (orange line) decreases in turn, and the collective steerability (pink lines) decays more slowly than its corresponding reduced bipartite steerability. Interestingly, even when the reduced bipartite steerability disappears completely, the collective steerability can still survive.

The effects of decoherence on the direction of collective steering were also studied. As shown in Fig. 5d, the PDC imposed on Charlie also leads to the asymmetric steering, i.e., $S_{k\vert \lbrace ij\rbrace }^{(n)}\ne S_{ \lbrace ij\rbrace \vert k}^{(n)}$. However, unlike the reduced bipartite steering case, there is no parameter window for one-way collective steering. And both $S_{k\vert \lbrace ij\rbrace }^{(n)}<1$ and $S_{ \lbrace ij\rbrace \vert k}^{(n)}<1$ hold once $d<1$. It means when the steered system is composed of two parties, they can be steered by the remaining party at the same time. Similarly, when the steering system contains two parties, they can steer the rest party simultaneously.

We further investigate how the shared steerability distributed among the parties from the perspective of Coffman–Kundu–Wootters (CKW) monogamy relation. In the case of three measurement settings, the monogamy relation involving either one steered party or two steered parties can be respectively expressed as^14^

2 $$\begin{aligned} \begin{aligned} \Delta S_{k\vert \lbrace ij\rbrace }^{(3)}\equiv S_{k\vert i}^{(3)}+S_{k\vert j}^{(3)}- 2S_{k\vert \lbrace ij\rbrace }^{(3)}>0,\\ \Delta S_{\lbrace ij\rbrace \vert k}^{(3)}\equiv S_{i\vert k}^{(3)}+S_{j\vert k}^{(3)}- 2S_{\lbrace ij\rbrace \vert k}^{(3)}>0, \end{aligned} \end{aligned}$$

where $i,j,k\in \lbrace A,B,C\rbrace$ in our case. We present the results in Fig. 5e,f. Clearly, as *d* increases, the monogamy relation Eq. (2) is always satisfied, which means two parties cannot independently demonstrate the steering of a third party. This is different from the above-mentioned results. In addition, we find there is a trade-off relation between $\Delta S_{k\vert \lbrace ij\rbrace }^{(3)}$ and $\Delta S_{\lbrace ij\rbrace \vert k}^{(3)}$, i.e., $\Delta S_{k\vert \lbrace ij\rbrace }^{(3)}$ increases with the decrease of the corresponding $\Delta S_{\lbrace ij\rbrace \vert k}^{(3)}$, and vice versa.

## Conclusions

In conclusion, we have theoretically investigated different nonlocal quantum correlations of genuine tripartite steering, reduced bipartite steering and collective steering of a tripartite generalized W state under three types of decoherence, amplitude damping, phase damping, and depolarizing. The region of decoherence strength and state parameters that each type of steering can survive were provided. Compared with ADC and DC, we found that these steering correlations were more resistant to PDC with increasing decoherence strength. In the case of tripartite, we observed that both 1SDI and 2SDI genuine tripartite steering under ADC behaved similarly to that under PDC when one of the state parameters was small, while their behaviors were similar to those under DC, when the state parameter became larger. In addition, we found they decayed faster than genuine tripartite entanglement and slower than genuine tripartite Bell nonlocality. In the case of bipartite, we investigated the dynamics of reduced bipartite steering and collective steering. The results show that reduced bipartite steering is more fragile in the presence of decoherence. With the average inference variance steering criterion, we further found that the direction of reduced bipartite steering and the symmetry of collective steering can be easily manipulated by changing the decoherence strength. What’s more, we considered the two steered parties situation. It has clearly shown that not only one party can be steered by a group system, but also two parties can be steered by a single system. However, in the view of CKW monogamy relation, we found two parties cannot independently demonstrate steering of a third system, showing a completely different conclusion from the average inference variance steering criterion.

Therefore, our findings provide clear theoretical evidence for the dynamic difference of steering correlations in the presence of decoherence. We also indicate the structural difference between various nonlocal quantum correlations and the efficiency of different steering criteria. Considering the fundamental and practical importance of quantum steering in quantum information science, our results not only provide an in-depth understanding of decoherence mechanism, but also inspire various applications of quantum information.

## Methods

In this paper, genuine tripartite steering, genuine tripartite entanglement, and genuine tripartite Bell nonlocality are demonstrated by violating the corresponding inequalities, respectively.

For the general three-qubit W state in the 1SDI scenario, the experimentally testable inequality for demonstrating genuine tripartite steering from Alice to Bob and Charlie is given by^18^

3 $$\begin{aligned} \begin{aligned} W_{1}&=1+0.4405(\langle \sigma _{z}^{B} \rangle + \langle \sigma _{z}^{C}\rangle )-0.0037\langle \sigma _{z}^{B}\sigma _{z}^{C}\rangle -0.1570( \langle \sigma _{x}^{B}\sigma _{x}^{C}\rangle +\langle \sigma _{y}^{B}\sigma _{y}^{C}\rangle +\langle A_{3}\sigma _{x}^{B}\sigma _{x}^{C}\rangle +\langle A_{3}\sigma _{y}^{B}\sigma _{y}^{C}\rangle )&\\&\quad +0.2424(\langle A_{3} \rangle +\langle A_{3}\sigma _{z}^{B}\sigma _{z}^{C}\rangle )+0.1848(\langle A_{3}Z_{B}\rangle +\langle A_{3}Z_{C}\rangle )-0.2533(\langle A_{1}\sigma _{x}^{B}\rangle +\langle A_{1}\sigma _{x}^{C}\rangle +\langle A_{2}\sigma _{y}^{B}\rangle +\langle A_{2}\sigma _{y}^{C}\rangle&\\&\quad +\langle A_{1}\sigma _{x}^{B}\sigma _{z}^{C}\rangle +\langle A_{1}\sigma _{z}^{B}\sigma _{x}^{C}\rangle )+\langle A_{2}\sigma _{y}^{B}\sigma _{z}^{C}\rangle +\langle A_{2}\sigma _{z}^{B}\sigma _{y}^{C}\rangle )\ge 0, \end{aligned} \end{aligned}$$

with $\lbrace A_{1},A_{2},A_{3}\rbrace$ being the observables associated with Alice’s measurements. $\langle \cdot \rangle$ denotes the expected value of a corresponding observable. Violation of this inequality implies genuine tripartite steering from Alice to Bob and Charlie. The maximum quantum violation is $-0.759$, which can be obtained only when Alice, Bob and Charlie share a pure W state ($\alpha =\beta =1/\sqrt{3})$ and Alice adopts the optimal measurement settings $\lbrace \sigma _{x},\sigma _{y},\sigma _{z}\rbrace$.

Similarly, for the general three-qubit W state in the 2SDI scenario, the genuine tripartite steering from Alice and Bob to Charlie can be detected by violating the following inequality^18^

4 $$\begin{aligned} \begin{aligned} W_{2}&=1+0.2517(\langle A_{3} \rangle + \langle B_{3}\rangle )+0.3520\langle \sigma _{z}^{C} \rangle -0.1112(\langle A_{1}\sigma _{x}^{C}\rangle +\langle A_{2}\sigma _{y}^{C}\rangle +\langle B_{1}\sigma _{x}^{C}\rangle +\langle B_{2}\sigma _{y}^{C}\rangle )+0.1296(\langle A_{3}\sigma _{z}^{C}\rangle +\langle B_{3}\sigma _{z}^{C}\rangle )&\\&\quad -0.1943(\langle A_{1}B_{1}\rangle +\langle A_{2}B_{2}\rangle )+0.2277\langle A_{3}B_{3}\rangle -0.1590(\langle A_{1}B_{1}\sigma _{z}^{C}\rangle +\langle A_{2}B_{2}\sigma _{z}^{C}\rangle )+0.2228\langle A_{3}B_{3}\sigma _{z}^{C}\rangle&\\&\quad -0.2298(\langle A_{1}B_{3}\sigma _{x}^{C}\rangle +\langle A_{2}B_{3}\sigma _{y}^{C}\rangle +\langle A_{3}B_{1}\sigma _{x}^{C}\rangle +\langle A_{3}B_{2}\sigma _{y}^{C}\rangle ) \ge 0, \end{aligned} \end{aligned}$$

with $\lbrace A_{1},A_{2},A_{3}\rbrace$ and $\lbrace B_{1},B_{2},B_{3}\rbrace$ being the observables associated with Alice’s and Bob’s measurements, respectively. The maximum quantum violation is $-0.480$, which can be obtained only when Alice, Bob and Charlie share a pure W state ($\alpha =\beta =1/\sqrt{3})$ and Alice and Bob adopt the optimal measurement settings $\lbrace \sigma _{x},\sigma _{y},\sigma _{z}\rbrace$.

The genuine entanglement of the three-qubit W state can be detected by violating the inequality in terms of the matrix elements, which can be written as^67,68^

5 $$\begin{aligned} \begin{aligned}{}&K_{W}= |\rho _{23}|+|\rho _{25}|+|\rho _{35}|-\sqrt{\rho _{11}\rho _{44}}-\sqrt{\rho _{11}\rho _{66}}-\sqrt{\rho _{11}\rho _{77}}-1/2(\rho _{22}+\rho _{33}+\rho _{55})\le 0. \end{aligned} \end{aligned}$$

If a state $$\varepsilon (\rho _{ABC})$$ gives $$ K_{W}>0$$, then genuine entanglement is certified. And the genuine Bell nonlocality is verified by violating the Svetlichny inequality^69^

6 $$\begin{aligned} \begin{aligned}{}&SI=\langle A_{1} B_{1}C_{2}\rangle -\langle A_{1} B_{2}C_{1}\rangle +\langle A_{2} B_{1}C_{1}\rangle +\langle A_{2} B_{2}C_{2}\rangle +\langle A_{2} B_{2}C_{1}\rangle -\langle A_{2} B_{1}C_{2}\rangle +\langle A_{1} B_{2}C_{2}\rangle +\langle A_{1} B_{1}C_{1}\rangle \le 4. \end{aligned} \end{aligned}$$

$\lbrace A_{1},A_{2}\rbrace$, $\lbrace B_{1},B_{2}\rbrace$ and $\lbrace C_{1},C_{2}\rbrace$ represent the measurement settings of Alice, Bob and Charlie, respectively. To maximize the violation, Alice and Charlie’s measurements are set as $\lbrace \sigma _{x},\sigma _{z}\rbrace$, Bob’s are set as $\lbrace (\sigma _{x}+\sigma _{z})/\sqrt{2}, (\sigma _{x}-\sigma _{z})/\sqrt{2}\rbrace$.

Here, we employ a steering criterion based on average inference variance to test the reduced bipartite steering and the collective steering. In the case of party *i* steers party *j*, the steering parameter has the following form^14,19^

7 $$\begin{aligned} S_{j\vert i}^{(n)}\equiv \dfrac{1}{C^{n}} \sum _{m=1}^{n} (\Delta _{\text{inf}}\sigma _{m}^{j}\vert \sigma _{m}^{i})^{2}, \end{aligned}$$

where $i,j\in \lbrace A, B,C\rbrace$, *n* is the number of the measurement settings. $\sigma _{m}^{i}$ and $\sigma _{m}^{j}$ represent the *m*-th measurement setting for party *i* and party *j*, respectively. $S_{j\vert i}^{(n)}<1$ indicates party *j* can be steered by party *i*. The amount of violation increases with *n*, i.e., for a larger *n*, a larger set of steerable states can be captured. To compare with the dynamical behaviors of genuine tripartite steering, we take $n=3$. In this case, $C^{n}=2$. And the optimal measurement settings of party *i* and party *j* are both set as $\{\sigma _{x},\sigma _{y},\sigma _{z}\}$.

By replacing single steering party with steering groups, the *n*-setting steerability parameter from the group parties *i* and *j* to party *k* can be written as

8 $$\begin{aligned} S_{k\vert \lbrace ij\rbrace }^{(n)}\equiv \dfrac{1}{C^{n}} \sum _{m=1}^{n} (\Delta _{\text{inf}}\sigma _{m}^{k}\vert \sigma _{m}^{i}\sigma _{m}^{j})^{2}, \end{aligned}$$

where $i,j,k\in \lbrace A, B,C\rbrace$. $\sigma _{m}^{i}$, $\sigma _{m}^{j}$ and $\sigma _{m}^{k}$ correspond to the *m*-th measurement setting of parties *i*, *j* and *k*, respectively. Similarly, the *n*-setting steering parameter from party *k* to the group parties *i* and *j* can be defined as $S_{ \lbrace ij\rbrace \vert k}^{(n)}\equiv \dfrac{1}{C^{n}} \sum _{m=1}^{n} (\Delta _{\text{inf}}\sigma _{m}^{i}\sigma _{m}^{j}\vert \sigma _{m}^{k})^{2}$. $S_{k\vert \lbrace ij\rbrace }^{(n)}<1$ and $S_{ \lbrace ij\rbrace \vert k}^{(n)}<1$ indicate the existence of the corresponding types of collective steering. Again, we mainly consider the case of $n=3$. To minimize $S_{k\vert \lbrace ij\rbrace }^{(3)}$ and $S_{ \lbrace ij\rbrace \vert k}^{(3)}$, the measurement directions of Alice, Bob, Charlie are set as $\lbrace \sigma _{x},\sigma _{y},\sigma _{z}\rbrace$.

## Supplementary Information

Supplementary Information.

## Data Availability

The datasets used and analysed during the current study available from the corresponding author on reasonable request.
