# Supplementary material for: Dynamics of multipartite quantum steering for different types of decoherence channels
Source: Sci Rep. 2023 Mar 7;13:3798. doi: 10.1038/s41598-023-30869-5 (PMC9992683; doi:10.1038/s41598-023-30869-5)
Supplement: Supplementary file 1 — Supplementary Information. [file 41598_2023_30869_MOESM1_ESM.pdf]

Supplementary information for Dynamics of multipartite quantum steering for different types of decoherence channels

Wei-Chen Li[1], *Ya Xiao*[1], *Xing-Hong Han*[1], *Xuan Fan*[1], *Xiao-Bing Hei*[1], and *Yong-Jian Gu*[1]

[1]*College of Physics and Optoelectronic Engineering, Ocean University of China, Qingdao* 266100, *People's Republic of China*

◇◇

I. DENSITY MATRIX ELEMENTS OF DIFFERENT DECOHERENCE CHANNELS

Decoherence caused by an unavoidable interaction between a system and environment can be expressed by the corresponding Kraus operations acting on the system state. As mentioned in the main text, given a generalized three-qubit W state ρ_{gW}, when only Charlie's qubit under a quantum channel with the decoherence strength d, the state ρ_{gW} evolves to $\varepsilon(\rho_{ABC}) = \sum_{m=1}^{n}(I^A \otimes I^B \otimes K_m^C)\rho_{gW}(I^A \otimes I^B \otimes K_m^C)^\dagger$. With the corresponding Kraus operators shown in Table I in the main text, the nonzero matrix elements ρ_{ij} of the evolved state in the amplitude damping channel becomes

$$\begin{aligned} &\rho_{11} = \alpha^2 d, \quad \rho_{22} = \alpha^2(1-d), \quad \rho_{23} = \rho_{32} = \alpha\beta\sqrt{1-d}, \\ &\rho_{33} = \beta^2, \quad \rho_{25} = \rho_{52} = \alpha\sqrt{1-d}\sqrt{1-\alpha^2-\beta^2}, \\ &\rho_{35} = \rho_{53} = \beta\sqrt{1-\alpha^2-\beta^2}, \quad \rho_{55} = 1-\alpha^2-\beta^2, \end{aligned} \tag{1}$$

the nonzero matrix elements ρ_{ij} of the evolved state in the phase damping channel becomes

$$\begin{aligned} &\rho_{22} = \alpha^2, \quad \rho_{23} = \rho_{32} = \alpha\beta\sqrt{1-d}, \\ &\rho_{33} = \beta^2, \quad \rho_{25} = \rho_{52} = \alpha\sqrt{1-d}\sqrt{1-\alpha^2-\beta^2}, \\ &\rho_{35} = \rho_{53} = \beta\sqrt{1-\alpha^2-\beta^2}, \quad \rho_{55} = 1-\alpha^2-\beta^2, \end{aligned} \tag{2}$$

and the nonzero matrix elements ρ_{ij} of the evolved state in the depolarizing channel becomes

$$\begin{aligned} &\rho_{11} = \alpha^2 d/2, \quad \rho_{22} = \alpha^2(2-d)/2, \quad \rho_{23} = \rho_{32} = \alpha\beta(1-d), \\ &\rho_{33} = \beta^2(2-d)/2, \quad \rho_{25} = \rho_{52} = \alpha(1-d)\sqrt{1-\alpha^2-\beta^2}, \\ &\rho_{35} = \rho_{53} = \beta(2-d)\sqrt{1-\alpha^2-\beta^2}/2, \quad \rho_{44} = \beta^2 d/2, \\ &\rho_{46} = \rho_{64} = \beta d\sqrt{1-\alpha^2-\beta^2}/2, \\ &\rho_{55} = (2-d)\left(1-\alpha^2-\beta^2\right)/2, \quad \rho_{66} = d\left(1-\alpha^2-\beta^2\right)/2. \end{aligned} \tag{3}$$

In addition, the reduced state between Alice and Bob, Alice and Charlie, Bob and Charlie can be obtained by taking the partial trace of the density matrix $\varepsilon(\rho_{ABC})$, i.e., $\varepsilon(\rho_{AB}) = \mathrm{Tr}_C[\varepsilon(\rho_{ABC})]$, $\varepsilon(\rho_{AC}) = \mathrm{Tr}_B[\varepsilon(\rho_{ABC})]$, and $\varepsilon(\rho_{BC}) = \mathrm{Tr}_A[\varepsilon(\rho_{ABC})]$. The nonzero matrix elements ρ_{ij} of the state $\varepsilon(\rho_{AB})$ in the phase damping channel becomes

$$\rho_{11} = \alpha^2, \quad \rho_{22} = \beta^2, \quad \rho_{33} = 1-\alpha^2-\beta^2, \quad \rho_{23} = \rho_{32} = \beta\sqrt{1-\alpha^2-\beta^2}. \tag{4}$$

The nonzero matrix elements ρ_{ij} of the state $\varepsilon(\rho_{AC})$ in the phase damping channel becomes

$$\rho_{11}=\beta^2,\quad \rho_{22}=\alpha^2,\quad \rho_{33}=1-\alpha^2-\beta^2,\quad \rho_{23}=\rho_{32}=\alpha\sqrt{1-d}\sqrt{1-\alpha^2-\beta^2}. \tag{5}$$

The nonzero matrix elements ρ_{ij} of the state $\varepsilon(\rho_{BC})$ in the phase damping channel becomes

$$\rho_{11}=1-\alpha^2-\beta^2,\quad \rho_{22}=\alpha^2,\quad \rho_{33}=\beta^2,\quad \rho_{23}=\rho_{32}=\alpha\beta\sqrt{1-d}. \tag{6}$$

II. STEERING PARAMETERS OF REDUCED BIPARTITE STEERING

For the generalized three-qubit W state considered in our work, the reduced bipartite steering parameters $S^{(3)}_{B|A}$ and $S^{(3)}_{A|B}$ between Alice and Bob in the phase damping channel can be expressed as

$$\begin{aligned} S^{(3)}_{A|B} &= \frac{\left(r^2-1\right)r^2\left(r^2\cos(4\theta)+\left(6-4r^2\right)\cos(2\theta)+3r^2-2\right)}{2r^2\sin^2\theta-2}+1,\\ S^{(3)}_{B|A} &= \frac{1}{4}\left(8r^4-r^2\left(\cos(4\theta)+8\left(r^2-1\right)\cos(2\theta)\right)-7r^2+4\right); \end{aligned} \tag{7}$$

the steering parameters $S^{(3)}_{C|A}$ and $S^{(3)}_{A|C}$ between Alice and Charlie in the phase damping channel can be expressed as

$$\begin{aligned} S^{(3)}_{A|C} &= -4dr^2\left(r^2-1\right)\cos^2\theta+\frac{3r^6-5r^4+3r^2+\left(r^2-1\right)r^4\cos(4\theta)+\left(4r^4-10r^2+7\right)r^2\cos(2\theta)-2}{2r^2\cos^2\theta-2},\\ S^{(3)}_{C|A} &= -4dr^2\left(r^2-1\right)\cos^2\theta+\frac{1}{4}\left(8r^4+8\left(r^2-1\right)r^2\cos(2\theta)-r^2\cos(4\theta)-7r^2+4\right); \end{aligned} \tag{8}$$

and the steering parameters $S^{(3)}_{C|B}$ and $S^{(3)}_{B|C}$ between Bob and Charlie in the phase damping channel can be expressed as

$$\begin{aligned} S^{(3)}_{B|C} &= (d-1)r^4\sin^2(2\theta)+2r^2\sin^2\theta+\frac{2r^4\sin^4\theta}{r^2\cos^2\theta-1}+1,\\ S^{(3)}_{C|B} &= (d-1)r^4\sin^2(2\theta)+2r^2\cos^2\theta+\frac{2r^4\cos^4\theta}{r^2\sin^2\theta-1}+1. \end{aligned} \tag{9}$$

Obviously, the coefficients of decoherence strength d in $S^{(3)}_{j|i}$ and $S^{(3)}_{i|j}$ are always the same, which means as d increases, the reduced bipartite steerability from party i to party j decreases at the same rate as that from party j to party i.
